# Body Mass Index and 1-Year Unplanned Readmission in Chinese Patients with Acute Myocardial Infarction: A Retrospective Cohort Study

**DOI:** 10.1155/2020/4158209

**Published:** 2020-02-18

**Authors:** Dandan Sun, Qingyun Zhang, Wei Li, Haichen Wang

**Affiliations:** ^1^Department of Cardiology of Affiliated Hospital of Jining Medical University, 89# Guhuai Road, Rencheng District, Jining 272000, Shandong Province, China; ^2^Nursing Department of Affiliated Hospital of Jining Medical University, 89# Guhuai Road, Rencheng District, Jining 272000, Shandong Province, China; ^3^Office of Party Committee of Affiliated Hospital of Jining Medical University, 89# Guhuai Road, Rencheng District, Jining 272000, Shandong Province, China

## Abstract

**Background:**

Evidence regarding the relationship between body mass index (BMI) and 1-year unplanned readmission was limited. Therefore, the objective of this research is to investigate whether BMI was independently related to 1-year unplanned readmission in Chinese patients with acute myocardial infarction (AMI) after percutaneous transluminal coronary intervention (PCI) after adjusting for other covariates.

**Methods:**

The present study was a cohort study. A total of 214 participants with AMI after PCI were involved in a hospital in China from 1^st^ January 2017 to 1^st^ January 2018. The target independent variable and the dependent variable were BMI measured at baseline and 1-year unplanned readmission, respectively. Covariates involved in this study included age, gender, TC, triglyceride, HDL-C, LDL-C, PT, APTT, INR, creatinine, HGB, LVEF, discharge medication, marital status, educational level, COPD, diabetes mellitus, heart failure, history of ischemic stroke, history of hemorrhagic stroke, arrhythmia, and hypertension.

**Results:**

The average age of 172 selected participants was 60.2 ± 10.8 years old, and about 68.6% of them was male. The rate of readmission in patients with AMI was 26.14%. The result of fully adjusted binary logistic regression showed BMI was negatively associated with risk of readmission after adjusting confounders (hazard ratio (HR) = 1.1, 95% CI 0.93–1.29). Nonlinear relationship was detected between BMI and 1-year unplanned readmission, whose point was 29.3. The effect sizes and the confidence intervals of the left and right sides of inflection point were 0.9 (0.7–1.2, *P* for nonlinearity = 0.530) and 2.8 (1.3–5.8, *P* for nonlinearity = 0.530) and 2.8 (1.3–5.8,

**Conclusion:**

BMI has a nonlinear relationship with 1-year unplanned readmission in patients with myocardial infarction. The 1-year unplanned readmission rate of overweight patients (BMI > 29.3 kg/m^2^) has increased significantly. Obesity paradox does not exist in terms of readmission of Chinese patients with myocardial infarction after PCI.

## 1. Introduction

The treatment of acute myocardial infarction (AMI) has made great progress, especially when percutaneous transluminal coronary intervention (PCI) and other interventional methods are widely used [1, 2]. However, there are still about 22.0%–29.5% patients who readmitted after their first discharge [3–6]. Previous studies have shown that AMI patients with rehospitalization have a significantly poorer prognosis and increase the economic burden [7–11]. Therefore, a study on the risk factors for readmission of AMI patients after PCI is needed. BMI is one of the indicators for measuring obesity [12]. Its association with a variety of diseases has been confirmed, including coronary heart disease, diabetes, sudden death, stroke, and metabolic syndrome [13–18]. Although there are some studies about the association between BMI and readmission of AMI in western countries, they yield conflict results [19–21]. Because of the ethnic differences, Asians and Westerners have different physiques. In addition, there is currently limited evidence of a link between BMI and readmission of AMI in the Chinese population. Therefore, this study set out to investigate whether BMI was independently related to 1-year unplanned readmission in Chinese patients with AMI after PCI.

### 1.1. Participants and Methods

#### 1.1.1. Study Design

In the present study, a retrospective cohort study was performed to address the relationship between BMI and readmission of AMI patients. The target independent variable is BMI obtained at baseline. The dependent variable is 1-year unplanned readmission (dichotomous variable: 1 = readmission after PCI; 0 = non-readmission).

#### 1.1.2. Study Population

The data of participants of Chinese patients with newly-diagnosed AMI were nonselectively and consecutively collected from the Department of cardiology, Affiliated Hospital of Jining Medical University, Jining City, Shandong province, China. Our data did not include identifiable participants data for the purpose of safeguarding patient privacy. Data were compiled from the hospital electronic medical record system. Participants informed consent is not required in this study because of the nature of the retrospective cohort study. The hospital institutional review board approved this study.

The study was initially collected a total of 214 participants. Participants' entry time and deadline for inclusion were 1 January 2017 and 1 January 2018, respectively. The clinical diagnosis and treatment process of each participant is completely in accordance with the ESC Guidelines on ST-segment elevation myocardial infarction, 2017. Inclusion criteria were as follows: 1, patients who were diagnosed with AMI in the emergency department; 2, patients who underwent PCI operation through emergency green channel. Exclusion criteria were as follows: 1, patients who changed their phone number; 2, patients who were not connected to the phone; 3, refused to answer the question; 4, patients of complicated vascular disease with coronary artery bypass surgery.

#### 1.1.3. Variables

We obtained BMI at baseline and recorded as continuous variable. The detailed process is described as follows: BMI, which was defined as weight in kilograms divided by height in meters squared (kg/m^2^). The height and weight of the patient were measured by the nurse at the time of the first admission.

According to published guideline and researches, we obtained the final outcome variable (dichotomous variable). We extracted the data of patients who are rehospitalized with the same ID number from the electronic case data system of the Affiliated Hospital of Jining Medical University. If no information was found about the patient's unplanned readmission, the subjects were followed up by telephone because the possibility of changing the ID number or going to another hospital was not ruled out.

In this study, we included the following covariates that can be summarized as follows: (1) demographic data; (2) variables that can affect BMI or 1-year unplanned readmission reported by the previous literature; (3) based on our clinical experiences. Therefore, the following variables were used to construct the fully-adjusted model: (1) continuous variable: age, total cholesterol (TC), triglyceride, high-density lipoprotein C (HDL-C), low-density lipoprotein C (LDL-C), prothrombin time (PT), activated part of prothrombin time (APTT), international normalized ratio (INR), creatinine, hemoglobin (HGB), and left ventricular ejection fraction (LVEF) (obtained at baseline); (2) categorical variables: gender, discharge medications, marital status, educational level, chronic obstructive pulmonary disease (COPD), diabetes mellitus, heart failure, history of ischemic stroke, history of hemorrhagic stroke, arrhythmia, and hypertension (obtained at baseline).

#### 1.1.4. Treatment Protocol

Percutaneous coronary intervention was implemented in each of our participants. Each patient is given personalized medication by the doctor.

#### 1.1.5. Follow-Up Procedure

We performed the follow-up through the telephone inquiry. The cutoff date for participants follow-up was 31^st^, January, 2019. Follow-up data were managed by the first author. Follow-up data were stored in the Hospital electronic medical record system. Follow-up interval was 1 year. Monitoring indicators at each follow-up included the patients' readmission information.

#### 1.1.6. Statistical Analysis

We presented continuous variables by two forms. In the first form, we expressed continuous variables with normal distribution as mean ± standard deviation. In the second form, we presented continuous variables with Skewed distribution as medium (min, max). Categorical variables were expressed in frequency or as a percentage. We used *χ*^2^ (categorical variables), one-way ANOVA test (normal distribution), or Kruskal–Wallis H test (skewed distribution) to test for differences among different BMI groups (Tertial). The data analysis process of this study was based on three criteria: (1) what is the relationship between BMI and readmission of AMI patients (linear or nonlinear); (2) which factors modify or interfere with the relationship between BMI and readmission of AMI patients; and (3) adjust the interference factors or after the stratified analysis, what is the true relationship between BMI and readmission of AMI patients? Therefore, data analysis can be summarized in two steps. Step 1: univariate and multivariate binary logistic regression was employed. We constructed three models: model 1, no covariates were adjusted; model 2, only adjusted for sociodemographic data; model 3, model 2 and other covariates presented in Table 1. Step 2: to address for nonlinearity of BMI and 1-year unplanned readmission, a Cox proportional hazards regression model with cubic spline functions and smooth curve fitting (penalized spline method) were conducted. If nonlinearity was detected, we first calculated the inflection point using recursive algorithm and then constructed a two-piecewise binary logistic regression on both sides of the inflection point. In the end, which model was more suitable for fitting the association between target independent variable and outcome variable was mainly determined by the log likelihood ratio test. For continuous variable, we first converted it to a categorical variable according to the clinical cut point or tertial. Tests for effect modification for those of subgroup indicators were followed by the likelihood ration test. To ensure the robustness of data analysis, we performed a sensitivity analysis. We converted the BMI into a categorical variable and calculated the *P* value for trend. The purpose was to verify the results of BMI as the continuous variable and to observe the possibility of nonlinearity. All the analyses were performed with the statistical software packages R (http://www.R-project.org, the R Foundation) and EmpowerStats (http://www.empowerstats.com, X&Y Solutions, Inc, Boston, MA). *P* values less than 0.05 (two-sided) were considered statistically significant.

## 2. Results

### 2.1. Baseline Characteristics of Selected Participants

**Figure 1:**
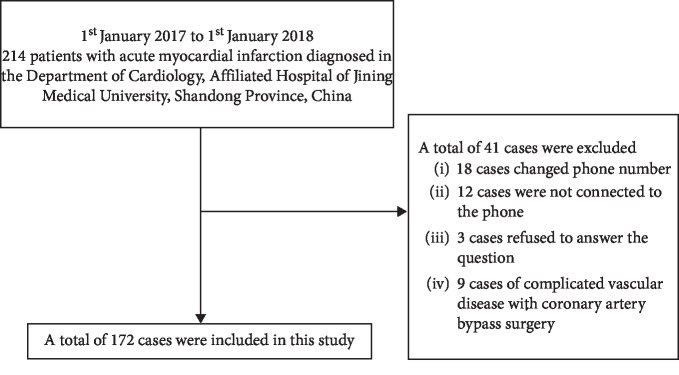
Inclusion/exclusion criteria.

A total of 172 participants were selected for the final data analysis after screening by inclusion and exclusion criteria (see Figure 1 for a flow chart). We showed baseline characteristics of these selected participants in Table 1 according to tertial of BMI. In general, the average age of the 172 selected participants was 60.2 ± 10.8 years old, and about 68.6% of them were male. The rate of readmission in patients with AMI was 26.14%. In the low, middle, and high BMI groups, the number of rehospitalized patients was 16 (27.59%), 12 (21.82%), and 17 (28.81%), respectively. No statistically significant differences were detected in age, gender, TC, LDL-C, PT, INR, creatinine, LVEF, discharge medications, educational level, COPD, diabetes mellitus, heart failure, history of ischemic stroke, history of hemorrhagic stroke, arrhythmia, and hypertension among different BMI groups (all *P* values >0.05). The subjects were divided into three equal parts according to the distribution of BMI. The BMI of the first group, the second group, and the third group was <23.6 kg/m^2^ (*n* = 58), 23.6–26.6 kg/m^2^ (*n* = 55), and >26.6 kg/m^2^ (*n* = 59), respectively. Participants with the highest group of BMI (T3) had the higher values in triglyceride and consisted of more married ones than those of the other groups. The opposite patterns were observed in HDL-C death of a spouse.

### 2.2. Univariate Analysis

We listed the results of univariate analyses in Table 2. By univariate binary logistic regression, we found that gender, TC, triglyceride, HDL-C, LDL-C, PT, APTT, INR, creatinine, HGB, LVEF, discharge medications, COPD, diabetes mellitus, heart failure, history of ischemic stroke, history of hemorrhagic stroke, arrhythmia, and hypertension were not associated with 1-year unplanned readmission. We also found that primary school (0.12, 0.04–0.08 vs ref), junior school (0.21, 0.08–0.59 vs ref), and high school/technical secondary school (0.08, 0.10–0.82 vs ref) were negatively associated with 1-year unplanned readmission. In contrast, univariate analysis showed that age (1.05, 1.01–1.08) was positively correlated with 1-year unplanned readmission.

### 2.3. Results of Unadjusted and Adjusted Binary Logistic Regression

In this study, we constructed three models to analyze the independent effects of BMI on 1-year unplanned readmission (univariate and multivariate binary logistic regression). The effect sizes (hazards ratio (HR)) and 95% confidence intervals are listed in Table 3. In the unadjusted model (model 1), the model-based effect size can be explained as the difference in 1 kg/m^2^ of BMI associated with risk of readmission. For example, the effect size of 1.02 for 1-year unplanned readmission in unadjusted model means that a difference in 1 kg/m^2^ of BMI is associated with increased 2% difference in risk of readmission (1.02, 95% CI 0.92–1.13). In the minimum-adjusted model (model 2), the BMI was increased by 1 kg/m^2^ and the risk of readmission increased by increase 6% (1.06, 95% CI 0.95–1.17). In the fully adjusted model (model 3) (adjusted all covariates presented in Table 1) for each additional 1 kg/m^2^ of BMI, the risk of readmission increased by increase 10% (1.1, 95% CI 0.93–1.29). For the purpose of sensitivity analysis, we converted the BMI from the continuous variable to categorical variable (Tertial of BMI); the *P* for trend of BMI with categorical variables in the fully-adjusted model was consistent with the result when BMI is a continuous variable. Besides, we also found the trend of the effect size in different BMI groups were nonequidistant.

### 2.4. Results of Nonlinearity of BMI and 1-Year Unplanned Readmission

**Figure 2:**
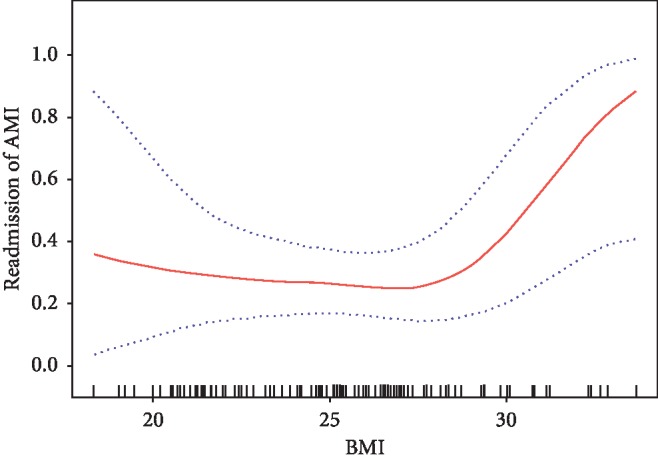
Association between BMI and readmission of AMI. A threshold, nonlinear association between BMI and readmission of AMI was found (*P*=0.022) in a generalized additive model (GAM). Solid rad line represents the smooth curve fit between variables. Blue bands represent the 95% of confidence interval from the fit, all adjusted for age, gender, TC, triglyceride, HDL-C, LDL-C, PT, APTT, INR, creatinine, HGB, LVEF, discharge medications, marital status, educational level, COPD, diabetes mellitus, heart failure, history of ischemic stroke, history of hemorrhagic stroke, arrhythmia, and hypertension.

In the present study, we analyzed the nonlinear relationship between BMI and 1-year unplanned readmission (Figure 2). Smooth curve and the result of the Cox proportional hazards regression model with cubic spline functions showed that the relationship between BMI, and BMI was nonlinear after adjusting for age, gender, TC, triglyceride, HDL-C, LDL-C, PT, APTT, INR, creatinine, HGB, discharge medications, marital status, educational level, COPD, diabetes mellitus, heart failure, history of ischemic stroke, history of hemorrhagic stroke, arrhythmia, and hypertension. We used both binary logistic regression and two-piecewise binary logistic regression to fit the association and select the best fit model based on *P* for the log likelihood ratio test.

Because the *P* for the log likelihood ratio test was less than 0.05, we chose two-piecewise binary logistic regression for fitting the association between BMI and 1-year unplanned readmission because it can accurately represent the relationship. By two-piecewise binary logistic regression and recursive algorithm, we calculated the inflection point was 29.3. On the left side of inflection point, the effect size and 95% CI were 0.9 and 0.7–1.2, respectively. On the right side of inflection point, the effect size and 95% CI were 2.8 and 1.3–5.8, respectively (Table 4).

## 3. Discussion

Our findings indicate BMI is negatively associated with 1-year unplanned readmission after adjusting other covariates. Besides, we also find the trend of the effect sizes on the left and right sides of the inflection point is not consistent (left 0.9 (95% CI 0.7–1.2); right 2.8 (95% CI 1.3–5.8)). This result suggests a threshold effect on the independent association between BMI and 1-year unplanned readmission.

Wang et al. [22] suggested that the risk of restenosis was lowest among underweight or normal weight patients and highest among severely obese patients in meta-analysis. However, there are also some other studies that are inconsistent with our findings. Paratz et al. [23] reported that obesity is not necessarily related with readmission in patients undergoing PCI. Akin et al. [24] showed that patients suffering from cardiogenic shock showed no impact of BMI on clinical endpoints. However, there are also some studies that showed that a higher BMI was not associated with worse outcomes of AMI patients after PCI, which was called obesity paradox [25, 26]. Whether the obesity paradox exists in Asian or Chinese patients still remains controversial [27]. We analyzed these studies that are inconsistent with our results, and we speculate that the reasons for the different results may be caused by the following factors: (1) the research population is different; these studies, which were inconsistent with our findings, were targeted at the USA; (2) these different conclusions do not clarify the nonlinear relationship; (3) compared with our work, these studies did not take into account the effect of triglyceride, HDL-C, PT, APTT, INR, creatinine, and HGB on the BMI and 1-year unplanned readmission relationships when adjusting covariates. However, the previous studies have confirmed that these variables are related to BMI or 1-year unplanned readmission [28−30]; (4) as with many other observational studies, reverse causality or residual confounding may potentially explain some findings.

The clinical value of this study is as follows: (1) to our best knowledge, it is the first time to observe the independent association between BMI and 1-year unplanned readmission in Chinese patients with AMI after PCI; (2) the findings of this study should be helpful for future research on the establishment of diagnostic or predictive models of 1-year unplanned readmission.

Our study has some strengths. (1) we address the nonlinearity in the present study and further explore this; (2) this study is an observational study and therefore susceptible to potential confounding; we used strict statistical adjustment to minimize residual confounders; (3) we handled target independent variable as both continuous variable and categorical variable. Such an approach can reduce the contingency in the data analysis and enhance the robustness of results.

There is some limitation in the present study including the following: (1) in this study, our research subjects are Chinese patients with AMI after PCI. Therefore, there is a certain deficiency in the universality and extrapolation of research. (2) Because we exclude patients of complicated vascular disease with coronary artery bypass surgery, the findings of this study cannot be used for these people.

## 4. Conclusions

In this retrospective study of Chinese patients with AMI after PCI, we found overweight patients (BMI > 29.3 kg/m^2^) were associated with increased odds of readmission. It is important for doctors to recommend their obese patients to lose weight. Prospective studies are needed to further examine the relationship between BMI and readmission to help guide management of Chinese AMI patients.

## Figures and Tables

**Table 1:** Baseline characteristics of participants.

BMI	T1	T2	T3	*P* value
*N*	58	55	59	
Age (years, mean ± sd)	62.8 ± 11.0	58.8 ± 9.7	59.0 ± 11.5	0.077
Gender, *n* (%)				0.355
Male	36 (62.1)	41 (74.5)	41 (69.5)	
Female	22 (37.9)	14 (25.5)	18 (30.5)	
TC (mmol/l, mean ± sd)	4.2 ± 1.0	4.3 ± 1.1	4.5 ± 1.2	0.517
Triglyceride (mmol/l, mean ± sd)	1.3 ± 0.7	1.5 ± 1.1	1.9 ± 1.5	0.017
HDL-C (mmol/l, mean ± sd)	1.1 ± 0.3	1.1 ± 0.2	1.0 ± 0.2	<0.001
LDL-C (mmol/l, mean ± sd)	2.6 ± 0.7	2.7 ± 0.9	2.8 ± 1.0	0.293
PT (sec, mean ± sd)	12.4 ± 4.3	11.3 ± 1.0	11.4 ± 1.0	0.069
APTT (sec, median, *Q*1–*Q*3)	25.8 (23.6–31.5)	24.1 (21.5–27.4)	24.4 (21.9–26.8)	0.015
INR (mean ± sd)	1.0 ± 0.4	1.2 ± 1.4	1.0 ± 0.1	0.463
Creatinine (*μ*mol/l, mean ± sd)	61.9 ± 15.0	68.9 ± 18.3	67.1 ± 16.3	0.072
HGB (g/l, mean ± sd)	135.5 ± 17.2	145.2 ± 17.6	143.4 ± 18.8	0.011
LVEF (%)	49.9 ± 8.5	49.3 ± 8.1	48.9 ± 8.6	0.938
Discharge medications, %				
*β*-blockers	43 (75.4)	40 (76.9)	48 (80.0)	0.834
Isosorbide mononitrate	29 (50.9)	25 (48.1)	31 (51.7)	0.925
Statins	51 (89.5)	51 (98.1)	54 (90.0)	0.155
Ticagrelor	27 (47.4)	32 (61.5)	33 (55.0)	0.331
ACEI	22 (38.6)	26 (50.0)	31 (51.7)	0.313
Clopidogrel	27 (47.4)	18 (34.6)	24 (40.0)	0.395
Aspirin	56 (98.2)	51 (98.1)	55 (91.7)	0.222
Marital status, *n* (%)				0.034
Married	48 (82.8)	53 (96.4)	58 (98.3)	
Unmarried	1 (1.7)	1 (1.8)	0 (0.0)	
Divorce	1 (1.7)	0 (0.0)	0 (0.0)	
Death of a spouse	8 (13.8)	1 (1.8)	1 (1.7)	
Educational level, *n* (%)				0.735
Illiteracy	11 (19.0)	5 (9.1)	9 (15.2)	
Primary school	17 (29.3)	15 (27.3)	14 (23.7)	
Junior school	18 (31.0)	18 (32.7)	20 (34.0)	
High school/technical secondary school	11 (19.0)	14 (25.5)	15 (25.4)	
Bachelor degree or above	1 (1.7)	3 (5.5)	1 (1.7)	
COPD, *n* (%)				0.781
No	55 (96.5)	51 (98.1)	58 (98.3)	
Yes	2 (3.5)	1 (1.9)	1 (1.7)	
Diabetes mellitus, *n* (%)				0.496
No	47 (81.0)	41 (74.5)	46 (78.0)	
Yes	11 (19.0)	14 (25.5)	13 (22.0)	
Heart failure, *n* (%)				0.636
No	52 (89.7)	52 (94.5)	53 (90.0)	
Yes	6 (10.3)	3 (5.5)	6 (10.0)	
History of ischemic stroke, *n* (%)				0.059
No	57 (98.3)	48 (87.3)	55 (93.2)	
Yes	1 (1.7)	7 (12.7)	4 (6.8)	
History of hemorrhagic stroke, *n* (%)				0.995
No	57 (98.3)	54 (98.2)	58 (98.3)	
Yes	1 (1.7)	1 (1.8)	1 (1.7)	
Arrhythmia, *n* (%)				0.619
No	50 (86.2)	49 (89.1)	54 (91.5)	
Yes	8 (14.8)	6 (10.9)	5 (8.5)	
Hypertension, *n* (%)				0.187
No	36 (62.1)	31 (56.4)	28 (47.5)	
Yes	22 (37.9)	24 (43.6)	31 (52.5)	

BMI, body mass index; TC, total cholesterol; HDL-C, high-density lipoprotein cholesterol; LDL-C, low-density lipoprotein cholesterol; PT, prothrombin time; APTT, activated partial thromboplastin time; INR, international normalized ratio; HBG, hemoglobin; COPD, chronic obstructive pulmonary disease.

**Table 2:** Univariate analysis for readmission of AMI.

Covariate	Statistics	HR (95% CI)	*P* value
Age (years, mean ± sd)	60.2 ± 10.8	1.05 (1.01, 1.08)	0.012
Gender, *n* (%)			
Male	118 (68.60)	Reference	
Female	54 (31.40)	1.05 (0.51, 2.16)	0.890
BMI (kg/m^2^, mean ± sd)	25.35 ± 3.37	1.02 (0.92, 1.13)	0.705
TC (mmol/l, mean ± sd)	4.33 ± 1.10	0.89 (0.65, 1.23)	0.491
Triglyceride (mmol/l, mean ± sd)	1.55 ± 1.15	0.67 (0.43, 1.06)	0.086
HDLC (mmol/l, mean ± sd)	1.05 ± 0.23	1.20 (0.27, 5.33)	0.086
LDL-C (mmol/l, mean ± sd)	2.69 ± 0.88	0.85 (0.57, 1.27)	0.417
PT (sec, mean ± sd)	11.67 ± 2.64	0.84 (0.60, 1.17)	0.552
APTT (sec, mean ± sd)	26.58 ± 10.03	0.99 (0.95, 1.03)	0.538
INR (mean ± sd)	1.04 ± 0.79	0.39 (0.04, 4.28)	0.444
Creatinine (sec, mean ± sd)	65.85 ± 16.75	1.01 (0.99, 1.03)	0.220
HGB (g/l, mean ± sd)	141.22 ± 18.30	0.98 (0.96, 1.00)	0.082
LVEF (%)	49.28 + 8.36	0.97 (0.93, 1.01)	0.117
Discharge medications, %			
*β*-blockers	134 (77.91)	0.74 (0.34, 1.62)	0.447
Isosorbide mononitrate	87 (50.58)	1.23 (0.62, 2.42)	0.550
Statins	159 (92.44)	1.24 (0.32, 4.70)	0.756
Ticagrelor	94 (54.65)	1.11 (0.56, 2.19)	0.765
ACEI	82 (47.67)	0.70 (0.36, 1.39)	0.313
Clopidogrel	70 (40.70)	1.03 (0.52, 2.06)	0.922
Aspirin	165 (95.93)	0.91 (0.17, 4.86)	0.911
Marital status, *n* (%)			
Married	161 (92.5)	Reference	
Unmarried	2 (1.1)	_§	0.989
Divorce	1 (0.6)	_§	0.992
Death of spouse	10 (5.7)	12.9 (2.6, 63.6)	0.002
Educational level, *n* (%)			
Illiteracy	25 (14.5)	Reference	
Primary school	46 (26.7)	0.1 (0.1, 0.4)	<0.001
Junior school	56 (32.6)	0.2 (0.1, 0.6)	0.003
High school/technical secondary school	40 (23.3)	0.3 (0.1, 0.8)	0.020
Bachelor degree or above	5 (2.9)	0.5 (0.1, 3.7)	0.517
COPD, *n* (%)			
No	168 (97.7)	Reference	
Yes	4 (2.3)	2.8 (0.4, 20.6)	0.307
Diabetes mellitus, *n* (%)			
No	134 (77.9)	Reference	
Yes	38 (22.1)	1.0 (0.4, 2.2)	0.946
Heart failure, *n* (%)			
No	157 (91.3)	Reference	
Yes	15 (8.7)	0.2 (0.0, 1.4)	0.100
History of ischemic stroke, *n* (%)			
No	160 (93.0)	Reference	
Yes	12 (7.0)	1.4 (0.4, 4.9)	0.594
History of hemorrhagic stroke, *n* (%)			
No	169 (98.3)	Reference	
Yes	3 (1.7)	5.7 (0.5, 64.2)	0.160
Arrhythmia, *n* (%)			
No	153 (89.0)	Reference	
Yes	19 (11.0)	1.0 (0.3, 2.9)	0.964
Hypertension, *n* (%)			
No	95 (55.2)	Reference	
Yes	77 (44.8)	1.2 (0.6, 2.3)	0.626

CI, confidence interval; HR, hazard ratio. _§: the model failed because of the small size.

**Table 3:** Relationship between BMI and readmission of AMI in different models.

Variable	Crude model		Model I		Model II	
	HR (95% CI)	*P* value	HR (95% CI)	*P* value	HR (95% CI)	*P* value
BMI (kg/m^2^)	1.02 (0.92, 1.13)	0.705	1.06 (0.95, 1.17)	0.314	1.13 (0.94, 1.36)	0.194
BMI						
T1	Reference		Reference		Reference	
T2	0.45 (0.05, 4.38)	0.532	0.38 (0.03, 6.45)	0.439	_§	0.873
T3	0.58 (0.05, 8.57)	0.592	0.72 (0.03, 12.86)	0.765	_§	0.873
*P* For trend		0.516		0.246		0.500

CI, confidence interval. Model I: adjusted for age and gender. Model II: adjusted for age, gender, TC, triglyceride, HDL-C, LDL-C, PT, APTT, INR, creatinine, HGB, LVEF, discharge medications, marital status, educational level, COPD, diabetes mellitus, heart failure, history of ischemic stroke, history of hemorrhagic stroke, arrhythmia, and hypertension._§: the model failed because of the small size.

**Table 4:** The results of BMI and readmission of AMI using two-piecewise linear regression.

Inflection point of BMI (kg/m^2^)	Effect size	95% CI	*P* value
＜29.3	0.9	0.7 to 1.2	0.530
≥29.3	2.8	1.3 to 5.8	0.008

Effect: readmission of AMI; cause: BMI. Adjusted: age, gender, TC, triglyceride, HDL-C, LDL-C, PT, APTT, INR, creatinine, HGB, LVEF, discharge medications, marital status, educational level, COPD, diabetes mellitus, heart failure, history of ischemic stroke, history of hemorrhagic stroke, arrhythmia, and hypertension.

## Data Availability

The data used to support the findings of this study are available from the corresponding author upon request.
